# A Novel Microcontroller-Based System for the Wheel-Running Activity in Mice

**DOI:** 10.1523/ENEURO.0260-21.2021

**Published:** 2021-11-18

**Authors:** Meina Zhu, Deepa Kamath Kasaragod, Kazuya Kikutani, Kei Taguchi, Hidenori Aizawa

**Affiliations:** 1 Department of Neurobiology, Graduate School of Biomedical and Health Sciences, Hiroshima University, Hiroshima 734-8553, Japan; 2 Department of Emergency and Critical Care Medicine, Graduate School of Biomedical and Health Sciences, Hiroshima University, Hiroshima 734-8553, Japan

**Keywords:** wheel running, circadian rhythm, open-source, mouse, microcontroller

## Abstract

Voluntary wheel-running activity is a way to assess rodents’ circadian rhythm and motivation for exercise. Deficits in these behaviors are implicated in the pathophysiology of sleep and psychiatric disorders. Limited space in animal facilities can hamper long-term monitoring of running wheel activity outside of the home cage. To address this issue, we provide a stand-alone solution to monitor the wheel-running activity of mice in their home cage. This system, named the wheel-running activity acquisition (WRAQ) system, is based on a microcontroller driven by a lithium polymer battery. With the WRAQ, we can record the wheel-running activity and illumination data for at least 30 d. Applying the WRAQ to an endotoxemia mouse model robustly detected the altered wheel-running activity and its recovery. With wireless data transfer capability extension, the system also allows for online monitoring and reporting of the circadian time (CT). We used the online monitoring of wheel-running activity with this extended WRAQ system and observed a significant shift of the active period in the circadian rhythm following a temporal chemogenetic activation of the suprachiasmatic nucleus (SCN)-subparaventricular zone (SPZ). Together, these findings indicate that the WRAQ system is a novel and cost-effective solution for the analysis of wheel-running activity in mice.

## Significance Statement

Wheel-running activity is commonly used to assess voluntary activity along with the circadian rhythm in rodents. Long-term recording of the activity within additional animal facility space and associated costs could hamper its use depending on the scale of the study. Here, we provide a cost-effective and stand-alone solution to measure wheel-running activity in the home cage following manipulation of the central nervous system. We used a microcontroller for an Internet of things solution to monitor behavioral and environmental data online. This novel approach may ultimately contribute to the real-time analysis of rodent behaviors during temporal genetic and pharmacological interventions.

## Introduction

Behavioral activity in the home cage is a basic phenotype analyzed in neuroscience animal studies. In particular, voluntary wheel-running activity changes are often associated with diseases in animal models (Siepka and Takahashi, 2005). For example, previous studies identified genes modulating the circadian rhythm of wheel-running activity by analyzing the activity of mutants (Takahashi, 2017). Despite requiring additional energy, access to a running wheel increases voluntary activity in most rodents, which might benefit phenotypic analyses by amplifying the differences in activity between control and mutant groups. This is especially the case when examining the circadian rhythm of wheel-running activity since voluntary activity is generally restricted to the active period of the circadian rhythm in rodents (Novak et al., 2012).

Equipment to measure wheel-running activity in mice is available commercially and mainly consists of a running wheel and a data acquisition system, which are placed inside and outside of the cage, respectively. Considering the high-density rack systems with smaller cages that house mice under specific pathogen-free conditions, equipment providing stand-alone operation and remote reporting of the acquired data online would be desirable for wheel-running activity analysis in mice. Streaming behavioral and environmental signals online would allow researchers to analyze the data in real time and manipulate the ongoing neural activity at specific circadian times (CTs) using genetic and pharmacological interventions.

We developed an open-source hardware system named wheel-running activity acquisition (WRAQ) based on a microcontroller recording mice’s voluntary wheel-running activity in their home cage. This system combines a low-profile running wheel with a reed switch and photoresistor for data acquisition, operating with a lithium polymer battery for at least 30 d and storing data on a microSD card for offline analysis. We validated the WRAQ system with a behavioral study by performing quantitative analysis of mice under different schedules of light entrainment and with systemic inflammation as a disease model. We further extended WRAQ to enable online monitoring of wheel-running activity using wireless recording capability. This capability allowed chemogenetic activation of specific neuronal pathways in a temporally specific manner.

## Materials and Methods

### Animals

All procedures involving animals were performed per the ARRIVE guidelines (https://arriveguidelines.org/arrive-guidelines) and were approved by the institutional experimental animal committee (A18-42-2 and A16-46-2). C57BL/6J mice (seven to eight weeks old, male; CLEA) were housed individually in plastic cages (CL-0104-2, width 225 × depth 338 × height 140 mm, CLEA) with free access to food and water, a 12/12 h light/dark cycle (LD), constant darkness (DD), or constant light illumination (LL; 120 lux), and regulated temperature and humidity in the range of 18–25°C and 30–60%, respectively. For the experiments conducted under DD or LL conditions, mice were housed individually three weeks before recording.

### Design of WRAQ and its extension with wireless data transfer capability (WRAQ-WiFi)

**Figure 1.**
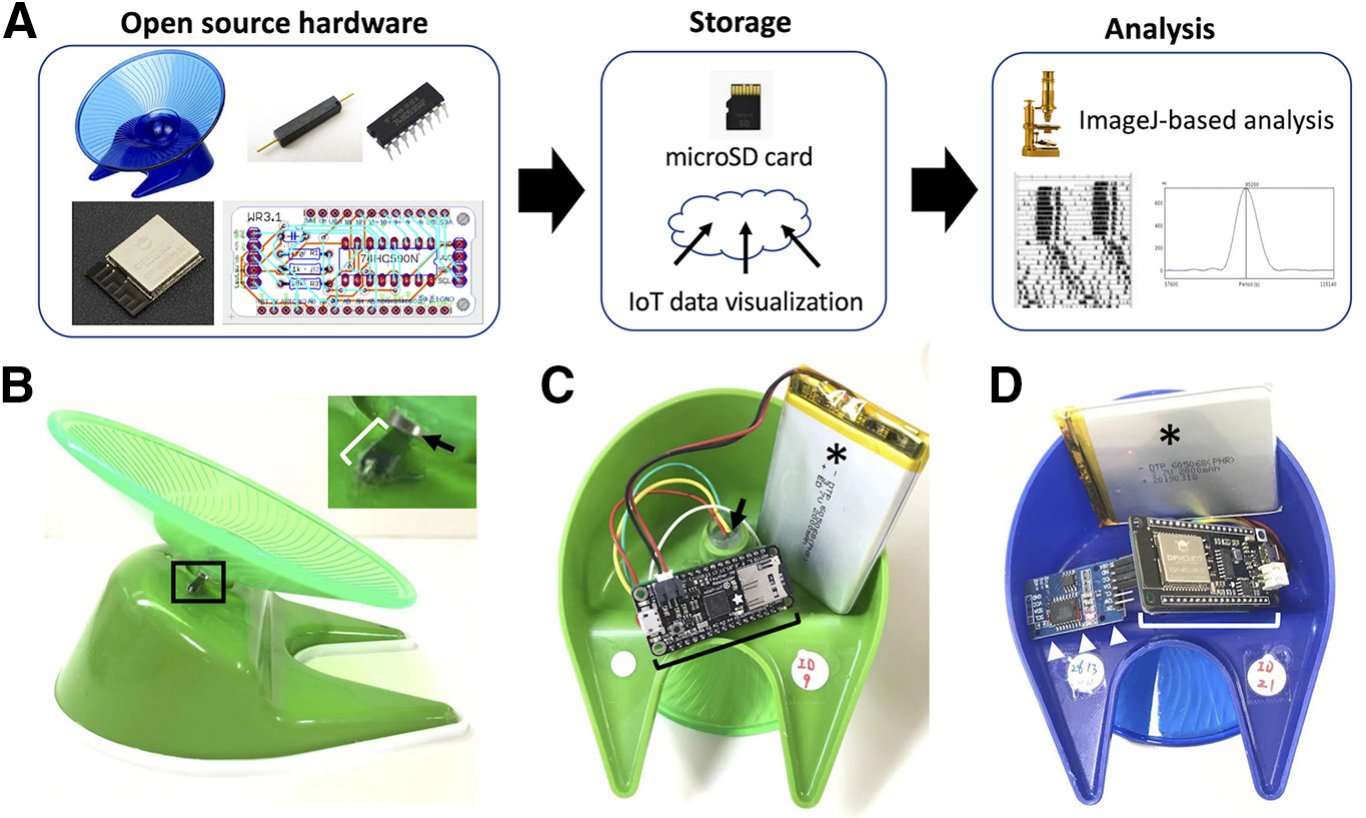
Overview of the WRAQ system. ***A***, Schematic showing the WRAQ system workflow. The WRAQ system acquires data using a low-profile running wheel detecting the number of revolutions and light intensity in the cage using a magnet-reed switch and photodiode sensor, respectively (left). Data are stored either to a microSD card or an online server via WiFi connection (middle). Analyses with actogram and periodogram are conducted offline using a software-based on ImageJ (right). ***B–D***, Side (***B***) or bottom views (***C***, ***D***) of the WRAQ (***B***, ***C***) and WRAQ-WiFi (***D***) showing the reed switch (bracket in ***B***) on the main body detecting the sweep by a magnet attached to the bottom of the rotating wheel (arrow in ***B***) and microcontrollers (brackets). The WRAQ and WRAQ-WiFi microcontrollers, i.e., Adalogger (bracket in ***C***) and FireBeetle ESP32 (bracket in ***D***), are connected to a lithium polymer battery (asterisks). Note that the photoresistor is encased in a silicon tube and placed at the center of the main body (arrow in ***C***). Inset shows a magnified view of a boxed area in ***B***. White arrowheads, real-time clock module.

**Figure 2.**
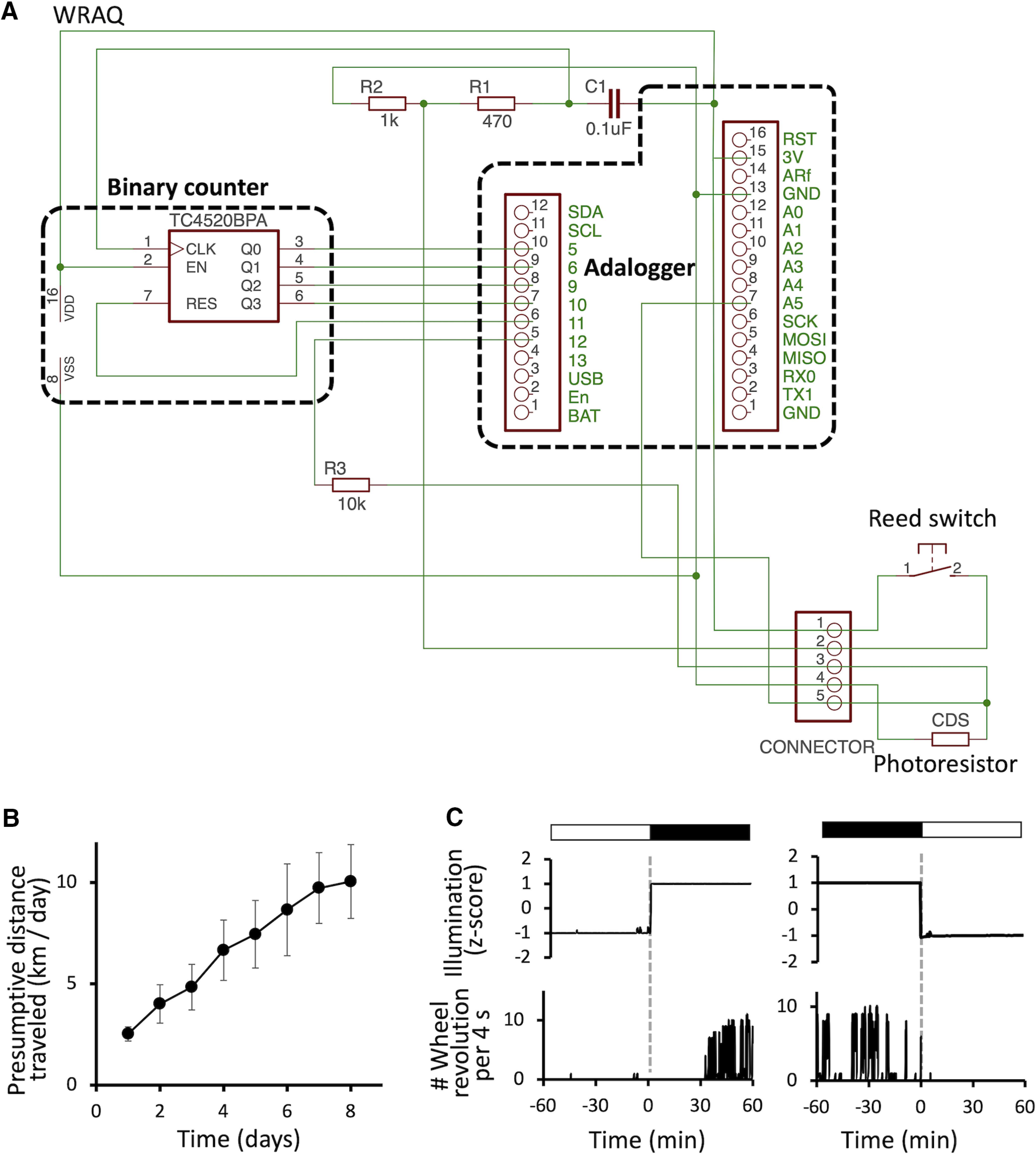
Simultaneous acquisition of the wheel revolution number and illumination data under light-dark light entrainment. ***A***, A schematic showing the hardware part of the WRAQ system primarily consists of a microcontroller for recording and system management (Adalogger) and a 4-bit binary counter which counts the number of wheel revolutions when the Adalogger is in deep sleep mode. ***B***, Line plot of the daily wheel-running activity during habituation to the WRAQ system. Values are represented as mean ± SEM. ***C***, Temporal changes of the normalized illumination (voltage across the photoresistor, upper traces) and the number of wheel revolutions per 4 s (bottom trances) are shown across the transitions between light and dark period (dashed gray lines). A, analog input; C, capacitor; CDS, cadmium sulfide photoresistor; CLK, clock input; EN, enabled; GND, ground; R, resistor; RES, reset; VDD, voltage drain; VSS, voltage source.

WRAQ was built based on a low-profile running wheel (flying saucer exercise wheel for small pets 5 inches, Ware Manufacturing Inc., width 5 inches × depth 5 inches × height 3.5 inches; Fig. 1*A*, left). A microcontroller managed data acquisition with a microSD card writer, Adafruit Feather M0 Adalogger, connected to a binary counter. The revolution of a small round magnet glued to the bottom of the wheel was detected by a reed switch attached to the main body of WRAQ (Fig. 1*B*). Upon sweeping the reed switch by the magnet, the number of revolutions was counted by the binary counter. Adalogger was in deep sleep mode to save power for long-term recording and woke up every 4 s to check the counter and voltage across the cadmium sulfide photoresistor (MI527, Macron International Group Ltd.) for illumination data. We stored the resultant data on a microSD card with the timestamps of the onboard real-time clock (Fig. 1*A*, middle). WRAQ was powered by either a lithium polymer battery (2000 mAh, Shenzhen Data Power Technology Ltd.) or a lithium AA battery (3.6V, Guangzhou Markyn Battery Co, Ltd.; Fig. 1*C*). The reed switch signal was connected to the binary counter through an RC lowpass filter to suppress chattering (Fig. 2*A*).

We extended WRAQ to WRAQ-WiFi, which enables online monitoring of ongoing wheel-running activity (Fig. 1*D*). The capability to upload data to the online data storage was implemented by WiFi connectivity using a built-in FireBeetle ESP32 IoT microcontroller (DFR0478, DFRobot), which replaced the Adalogger. In addition, we attached a real-time clock breakout board (catalog #3013, Adafruit Industries or zs-042, HiLetgo) based on the real-time clock DS3231 (Maxim Integrated), allowing ESP32 to access time stamps via an I^2^C protocol. We acquired the number of revolutions and the illumination data as in WRAQ. ESP32 was set to wake up from deep sleep mode and upload the data to the Ambient IoT data visualization cloud service (AmbientData Inc., https://ambidata.io/), enabling users to monitor the ongoing and collected data online (Fig. 1*A*, middle).

We inserted all the parts, except the magnet and reed switch, into the main body of the flying saucer, covered it with a 3D-printed plastic part, and sealed it with a peelable silicon adhesive (1690, Amon Industry Co, Ltd.).

### Analysis of the wheel-running activity data

ActogramJ software (https://bene51.github.io/ActogramJ/; Schmid et al., 2011), which is based on ImageJ (http://imagej.nih.gov/ij/; Schneider et al., 2012), was used for offline analysis of the wheel-running activity data retrieved from the microSD card (WRAQ) or downloaded from the cloud service (WRAQ-WiFi; Fig. 1*A*, right). The raw data were converted into the file readable by ActogramJ, in which the data were supposed to start at zeitgeber time (ZT)12, by custom-made python program (main_wraq2actj.py provided as Extended Data). The actogram and periodogram using Lomb–Scargle methods were calculated as described previously (Schmid et al., 2011).

### Treatment with lipopolysaccharide (LPS)

Three weeks after habituation under DD, mice were administered a single intraperitoneal injection of saline or LPS derived from *Escherichia coli* (O55:B5, L2880, Sigma-Aldrich) at a dose of 2.5 mg/kg. Their wheel-running activity was continuously recorded with the WRAQ system placed in their home cage across the LPS injection.

### Chemogenetic activation of the suprachiasmatic nucleus (SCN)-subparaventricular zone (SPZ)

We anesthetized mice with a mixture of ketamine (90 mg/kg) and xylazine (10 mg/kg) and immobilized it in a stereotaxic frame (SR6N, Narishige). Following a midline incision of the skin covering the skull, we made a burrhole to open a cranial window at 0.48 mm posterior and 0.15 mm lateral to the bregma over the SCN-SPZ (Franklin and Paxinos, 2008). A fine glass capillary was used to inject 0.05 μl of AAV8-hSyn-hM3D(Gq)-mCherry (2.5 × 10e12 gc/ml, catalog #50474, Addgene; RRID:Addgene_50474) or AAV8-CAG-GFP (2 × 10e12 vm/ml, UNC GTC Vector Core, University of North Carolina, Chapel Hill, NC) with a speed of 0.1 μl/min targeting the bilateral SCN-SPZ (5.8 and 5.7 mm deep from the pia mater). After closing the skin covering the cranial window by suture, the mouse was allowed to recover in its home cage for at least 7 d. Then, we administered a solution of clozapine N-oxide (CNO; 1 mg/kg, BML-NS105-0005, Enzo Life Sciences) by intraperitoneal injection to activate cells expressing hM3D.

### Data analysis

We converted illumination data recorded from the WRAQ and WRAQ-WiFi systems into a *z* score using each mean and standard deviation. The wheel revolutions were represented using 4-s (WRAQ) or 64-s (WRAQ-WiFi) bins. We discarded part of the raw data so it would start at ZT12 and then imported into ActogramJ to calculate a periodogram using the Lomb–Scargle method. Presumptive distance traveled was calculated by multiplying the number of wheel revolutions recorded by WRAQ or WRAQ-WiFi with the perimeter of the presumptive trace on the running wheel (25.12 cm). For analysis using periodogram, we excluded data with wheel-running activity under 20,000 revolutions per day.

### Immunohistochemistry

Two hours after intraperitoneal injection of CNO, mice with AAV8-hSyn-hM3D(Gq)-mCherry or AAV8-CAG-GFP were perfused transcardially using 4% paraformaldehyde (PFA) in 0.1 m PBS. After that, we dissected the brain and postfixed it in the same fixative overnight at 4°C. Then, 75-μm-thick coronal sections were cut using a vibratome (DTK-1500, Dosaka EM Co, Ltd.) from 0.1 to 0.9 mm posterior to the bregma (Franklin and Paxinos, 2008). Sections were washed with 0.5% PBS Triton X-100 and incubated in primary antibody against c-Fos (1:500, sc-271243, Santa Cruz Biotechnology) dissolved in 1% blocking reagent in 0.5% PBS Triton X-100 overnight at 4°C. Signal was visualized by a secondary antibody Alexa Fluor 488-conjugated donkey anti-mouse IgG (1:500, ab150105, Abcam plc.) or Alexa Fluor 594 AffiniPure donkey anti-mouse IgG (1:500, 715-585-150, Jackson ImmunoResearch) diluted in 0.5% PBS Triton X-100 overnight at 4°C. All sections were counterstained with 4′,6-diamidino-2-phenylindole (DAPI; 1 μg/ml, 422801, BioLegend), mounted with CC/Mount (Diagnostic BioSystems Inc.) and examined under a fluorescent microscope (MVX10, Olympus Corporation) or a laser scanning confocal microscope (FV1000, Olympus Corporation).

### Statistical analysis

Statistical analyses were performed using jamovi (version 1.1.9, https://www.jamovi.org). Comparisons between more than two groups were analyzed by one-way or repeated measure two-way ANOVA followed by Tukey’s HSD test for multiple comparisons. Pearson’s correlation analysis determined the correlation between the body weight and presumptive travel distance on the running wheel. Statistical significance was defined as a value of *p* < 0.05. Data are presented as mean ± SEM.

### Software accessibility

All the files for python program, bill of materials, microcontroller firmware, 3D-printed part, and printed circuit boards used in this study are available as Extended Data 1 and online (https://github.com/neurobio-hiroshima/WRAQ).

10.1523/ENEURO.0260-21.2021.ed1 Extended Data 1 Files used in the study. Download Extended Data 1, ZIP file.

## Results

### Simultaneous recording of the wheel-running activity and illumination using WRAQ

Mice in their home cage rotated the low-profile wheel of WRAQ already on the day of installation. The wheel revolutions gradually increased and plateaued around 7 d (Fig. 2*B*, *n* = 9 mice). We determined whether WRAQ could simultaneously detect wheel-running activity and illumination intensity changes in the home cage per the scheduled illumination for LD cycles. Resistance changes in photoresistors resulted in a persistent decrease and increased voltage detected by WRAQ during the light and dark periods, respectively (Fig. 2*C*, top). Consistent with the nocturnal behavior of mice, wheel-running activity exhibited an abrupt increase and decreased following the onset and end of the dark period, respectively (Fig. 2*C*, bottom). Considering that WRAQ sampled data every 4 s, these results indicate that WRAQ detected wheel-running activity of mice and the illumination intensity in their home cage with temporal precision.

### Long-term recording of the circadian rhythm in wheel-running activity under LD entrainment

**Figure 3.**
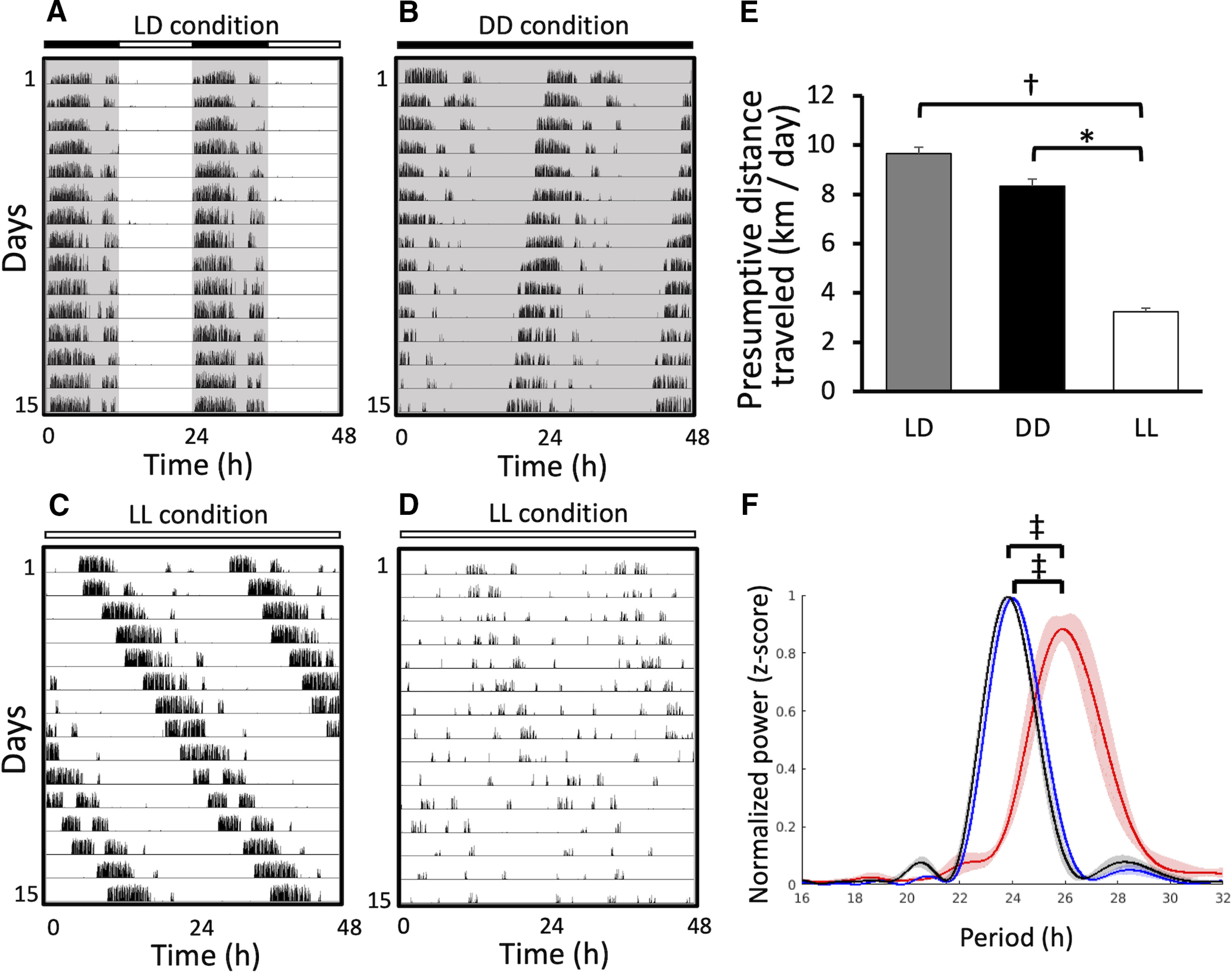
Wheel-running activity acquired by the WRAQ system under different entrainment schedules. ***A–D***, Actograms of mouse wheel-running activity under LD (***A***), DD (***B***), and LL (***C***, ***D***) conditions. ***E***, Bar graph of the presumptive distance traveled on the wheel for mice kept under LD (gray), DD (black), and LL (white) entrainment. ***F***, Line plots of the periodograms analyzing wheel-running activity recorded for 8 d under LD (blue, *n* = 10), DD (gray, *n* = 10), and LL (red, *n* = 7) based on the Lomb–Scargle method. Data are presented as mean (solid lines) ± SEM (error bars and shading). Contrasts are statistically significant differences of mean values between groups (repeated-measures ANOVA followed by Tukey’s *post hoc* test); *p < 0.05, †*p* < 0.01, ‡*p* < 0.001.

Next, we applied WRAQ to analyze the circadian rhythm of wheel-running activity in mice. Under a 12:12 h LD cycle, the number of wheel revolutions followed a diurnal rhythm with exclusive activity during the dark period (Fig. 3*A*). The active period started following the onset of the dark period. After a significant reduction of activity in the second half of the dark period, mice exhibited a shorter bout of wheel-running activity, which resulted in bimodal peaks of wheel-running activity, with distinct early and late-night activity bouts as reported previously (Pittendrigh and Daan, 1976).

Under altered schedules in light entrainment, particularly DD, WRAQ recorded the free-running in circadian rhythm with a shortening of the period. The resultant actogram exhibited a gradual advance of the onset of an active period on ZT (Fig. 3*B*). By contrast, LL led to variable changes in circadian rhythm, ranging from free-running with an elongated period (Fig. 3*C*) to an arrhythmic pattern (Fig. 3*D*). The LL condition also resulted in a significant reduction of the presumptive distance traveled compared with mice under LD or DD conditions (one-way ANOVA, *F*_(2,15.5)_ = 15.9, Tukey’s HSD test, *p* < 0.001, *n* = 9 mice for each group; Fig. 3*E*).

To measure the circadian rhythm in wheel-running activity, we subsequently calculated a periodogram using the ImageJ-based analysis software ActogramJ (Schmid et al., 2011). As an output of WRAQ, a comma-separated value file was imported into ActogramJ and analyzed using Lomb–Scargle methods. The results showed that the peak of the periodogram under LD was ∼24 h (mean peak value ± SEM, 24.0 ± 0.0844 h). DD tended to shorten the period (23.8 ± 0.0533 h), while LL elongated the period significantly (25.9 ± 0.2614 h, one-way ANOVA, *F*_(2,7.52)_ = 28.3, *p* < 0.001, Tukey’s HSD *post hoc* test, *p* < 0.001 for both LL vs DD and LL vs LD) with free-running along the circadian rhythm (Fig. 3*F*).

These results indicate that WRAQ is a useful tool for quantifying wheel-running activity behavioral determinants and can be integrated into an open-source analysis.

### Alteration and recovery of the wheel-running activity in a murine endotoxemia model

**Figure 4.**
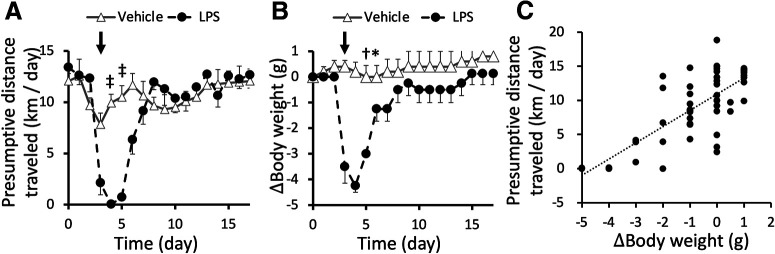
Transient suppression and recovery of wheel-running activity in a murine endotoxemia model. ***A***, ***B***, Line plots of daily wheel-running activity presented as the presumptive distance traveled (***A***) and relative body weight change from baseline (***B***) in mice with (filled circles with dashed lines) or without (triangles with solid lines) LPS treatment. Values are presented as mean ± SEM. Arrows indicate the timing of LPS injection. ***C***, Scatter plot of body weight change and presumptive distance traveled showing a positive correlation with statistical significance (Pearson’s correlation coefficient = 0.745, *p* < 0.001). Contrasts are statistically significant differences between groups based on a repeated measure two-way ANOVA followed by Tukey’s *post hoc* test; *p < 0.05, †*p* < 0.01, ‡*p* < 0.001.

Systemic administration of LPS has been used as a model of endotoxemia, which induces systemic inflammation (Beutler, 2000). As a previous study showed a significant reduction of mice locomotor activity in an open field arena 24 h after systemic LPS injection (Giga et al., 2021), we applied WRAQ to determine mouse behavior before and after LPS to evaluate its applicability to murine disease models. As compared with the behavior before LPS injection, intraperitoneal administration of 2.5 mg/kg LPS significantly reduced voluntary wheel-running activity (repeated-measures ANOVA, group × time interaction, *p* < 0.001, *F*_(17,153)_ = 8.95; Tukey’s *post hoc* test, *p* < 0.001 for vehicle vs LPS at days 4 and 5, *n* = 5 and 7 for vehicle and LPS groups, respectively; Fig. 4*A*). This change was accompanied by a transient reduction of body weight (repeated-measures ANOVA, group × time interaction, *p* < 0.001, *F*_(17,119)_ = 9.04; Tukey’s *post hoc* test, *p* < 0.001 for vehicle vs LPS at days 5 and 6; Fig. 4*B*). Indeed, analysis revealed that the body weight change was positively correlated with wheel-running activity (Pearson’s correlation coefficient = 0.745, *p* < 0.001; Fig. 4*C*), indicating that WRAQ detected a behavioral measure during endotoxemia inducing body weight loss. Intriguingly, long-term recording by WRAQ also unraveled a gradual recovery of wheel-running activity (Fig. 4*A*, black), further supporting the applicability of WRAQ to mouse models of diseases requiring longitudinal observation of long-lasting behaviors in the home cage.

### Chemogenetic activation of the SCN-SPZ shifts the onset of the active period under DD

**Figure 5.**
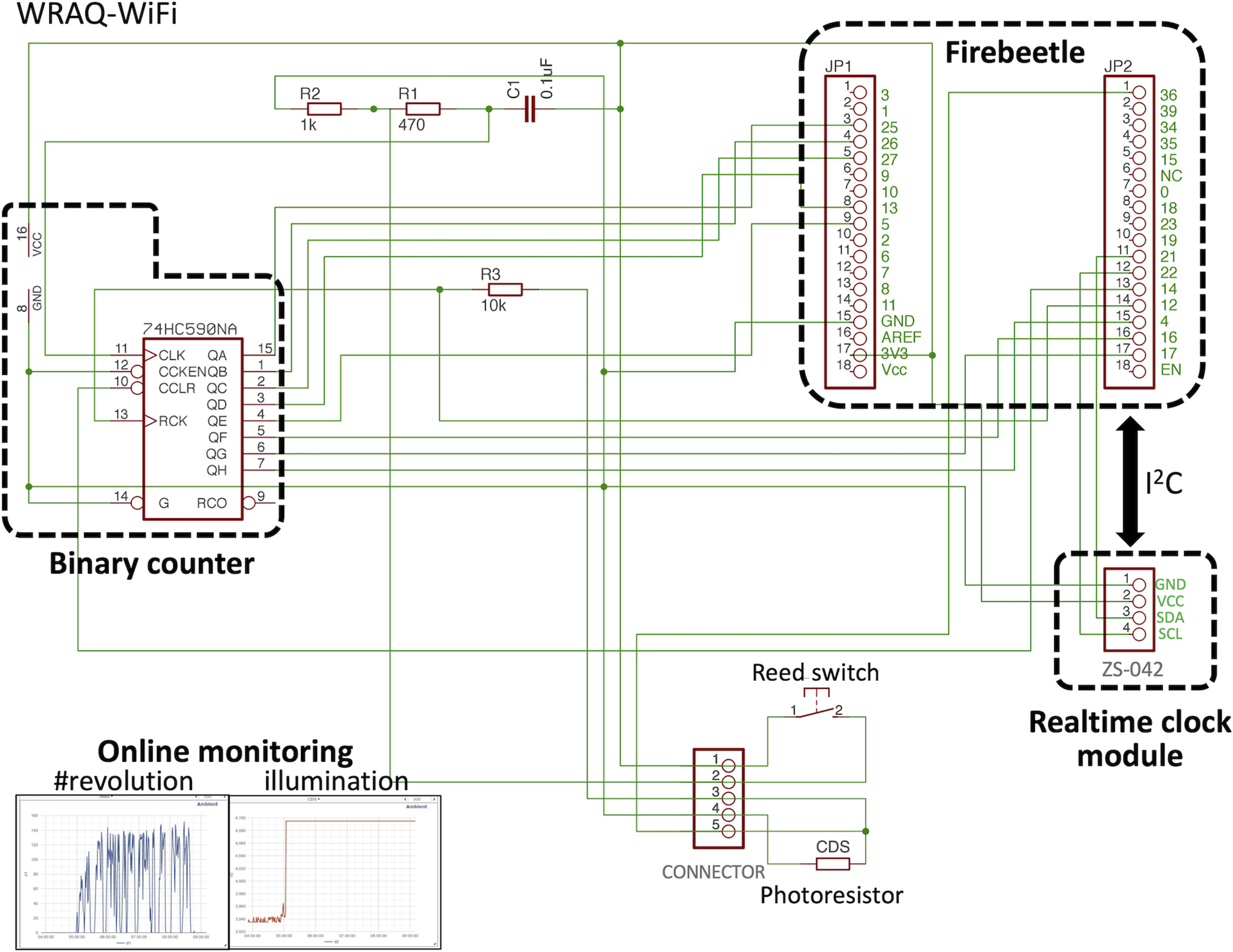
Wiring diagram for the WRAQ-WiFi. WRAQ-WiFi is based on ESP32 microcontroller implemented on a FireBeetle ESP32 board. The number of running wheel revolutions is measured by 8-bit counter 74HC590NA and transmitted to the cloud server and the timestamp (using real-time clock module) and illumination data in the home cage (based on change of photoresistor). Uploaded data are visualized on the online platform for real-time monitoring (panels at the lower-left corner). C, capacitor; CCLR; counter clear; CCKEN, counter clock enabled; CDS, cadmium sulfide photoresistor; CLK, clock input; G, ground; GND, ground; QA-QH, digital outputs from the binary counter; R, resistor; RCK, register clock; RES, reset; SDA, serial data line for I2C; SCL, serial clock line for I^2^C; VCC, voltage common collector.

Based on our success in acquiring longitudinal data of wheel-running activity, we extended our system capability to enable online monitoring via data uploading to a cloud server. Implementation of this version of WRAQ as IoT (WRAQ-WiFi) integrates ESP32 microcontroller with WiFi capability (Fig. 5). The uploaded data remained available while WRAQ-WiFi was under-recording (Fig. 5).

**Figure 6.**
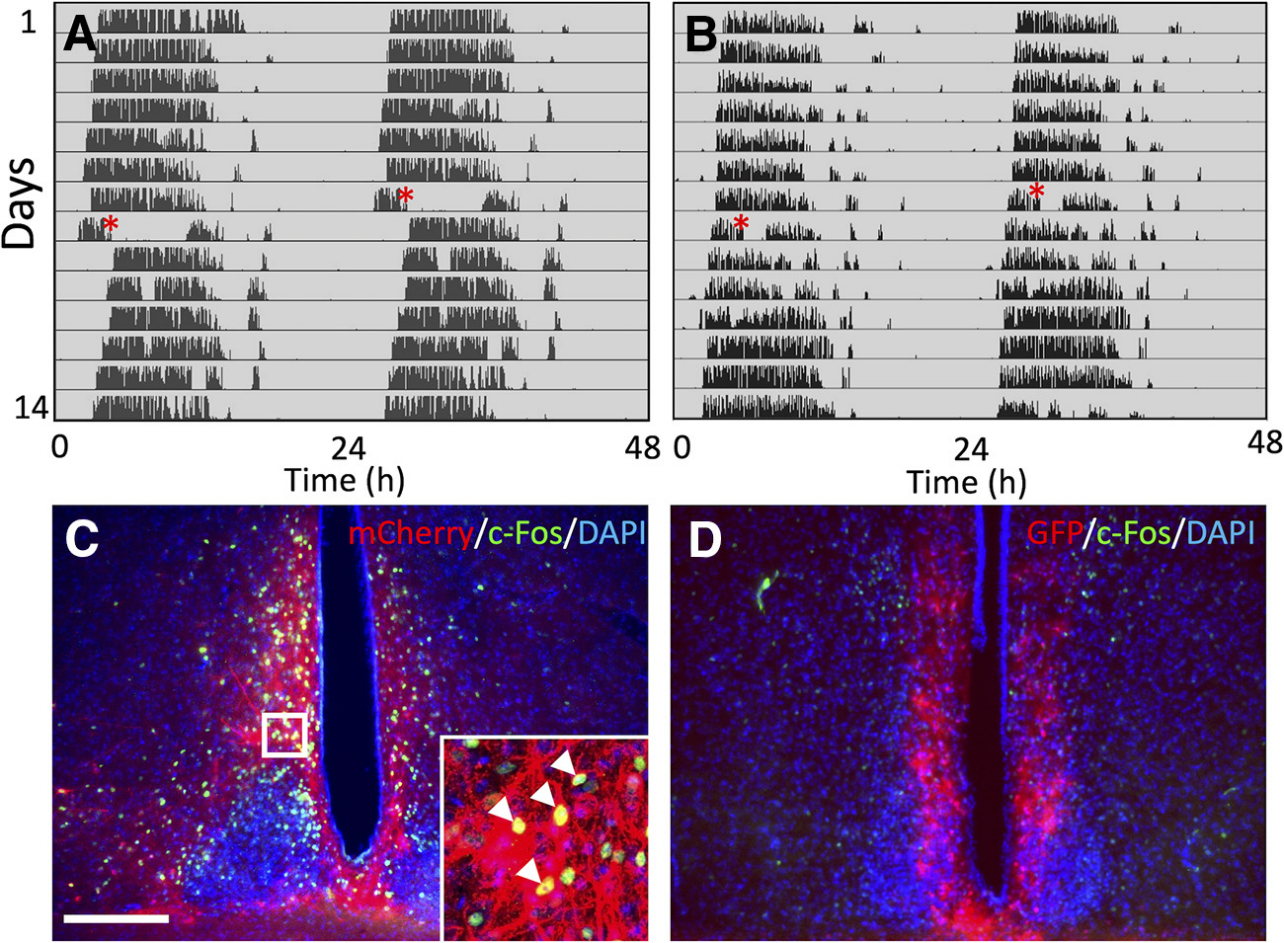
Temporarily specific neural activation patterns under the guidance of WRAQ-WiFi. ***A***, An actogram of mice receiving an injection of AAV8-Syn-hM3D-mCherry (***A***) or AAV8-CAG-GFP (***B***) to the SCN-SPZ under constant dark condition. On day 7, CNO was injected intraperitoneally at CT14, estimated based on the WRAQ-WiFi data available online (red asterisks in ***A***, ***B***). ***C***, ***D***, Coronal brain sections of the mouse used for behavioral analysis (***A***, ***B***) showing the localization of mCherry (red in ***C***), GFP (pseudocolored red in ***D***), and c-Fos (green in ***C***, ***D***) in the SCN-SPZ. Independent of the behavioral recording in ***A***, ***B***, the mice brains were fixed 2 h after the CNO injection. Sections were counterstained with DAPI (blue in ***C***, ***D***). An inset is a magnified view of the boxed area in ***C***. White arrowheads indicate the cells co-expressing mCherry (red) and c-Fos. Scale bar in ***C*** (applies to ***D***): 200 μm.

During free-running along the circadian rhythm of mice under DD, we applied WRAQ-WiFi to manipulate neuronal activity at a specific CT. Studies showed that SCN-SPZ acted as a master clock and a region relaying the circadian information from the SCN to other brain regions, respectively (Ibuka and Kawamura, 1975; Lu et al., 2001). We measured the wheel-running activity of a mouse targeted by AAV-hSyn-hM3D-mCherry to upregulate neuronal activity in the SCN-SPZ on systemic administration of CNO (Fig. 6*A*,*C*, *n* = 5 mice). We observed a significant increase of c-Fos-positive cells in the SCN-SPZ following intraperitoneal injection of CNO (Fig. 6*C*). Before CNO injection, the mouse exhibited free-running activity under DD (Fig. 6*A*). Under the guidance of WRAQ-WiFi, injection of CNO at CT14 (Fig. 6*A*, red asterisks) induced a significant shift in the onset of the active period lasting at least 7 d (Fig. 6*A*). In contrast, intraperitoneal injection of CNO to the mice expressing GFP in the SCN-SPZ did not induce any of these effects on the onset of active period (Fig. 6*B*) and c-Fos in SCN-SPZ (Fig. 6*D*) significantly (*n* = 5 mice).

These data reveal that online monitoring with the WRAQ-WiFi system enables studies requiring a temporally specific genetic or pharmacological intervention.

## Discussion

The present study demonstrated precise data acquisition of wheel-running activity along the circadian rhythm in mice within their home cage using the WRAQ system enabled by open-source hardware. The recorded data could be visualized offline and online when uploaded to the data visualization server in WRAQ-WiFi. Continuous monitoring of the wheel-running activity and circadian rhythm revealed altered activity and rhythms in mice exposed to systemic inflammation or a chemogenetic manipulation of a specific neuronal circuit. These results indicated that WRAQ is a novel tool that allows to explore the mechanisms underlying behaviors in murine disease models. We discuss below the utility of WRAQ and its limitation.

### Comparison with currently available technologies

A general configuration of systems recording wheel-running activity in rodents consists of microswitch and data acquisition board or interface with PC. Although communication between the running wheel with the switch and data acquisition parts can be wired or wireless, the presence of a data acquisition board or interface with PC can hamper their use in vivariums with limited space or high biosafety levels (Balcombe, 2006). WRAQ and WRAQ-WiFi store data on a built-in SD card or cloud server and work as a stand-alone device without requiring additional appendages, which allows their use in a wide variety of conditions generally encountered in mouse housing facilities. Because of its open-source nature, WRAQ-WiFi also has wide applicability for combination with other open-source IoT platforms such as ThingsBoard.

The size of the WRAQ system (width 5 inches × depth 5 inches × height 3.5 inches) is smaller than representative low-profile running wheels (ENV-047, Med Associate Inc.; width 6.1 inches × depth 6 inches × height 4 inches) used widely in neuroscience studies with mouse models. Since the latter low-profile mouse running wheel was used successfully in standard “shoebox” style individually ventilated cages (e.g., model 9, Thoren Caging Systems with width 7.70 inches × depth 12.17 inches × height 5.875 inches; Goh and Ladiges, 2015; Beeler and Burghardt, 2021), it is reasonable to think that our WRAQ fits a wide variety of cage systems.

Similar to other commercially available systems such as ClockLab (Actometrics, Co Ltd.), WRAQ/WRAQ-WiFi, built on open-source platform, is compatible with the recording of additional behaviors (e.g., general home cage activity, food and water consumption) and environmental data (e.g., temperature and humidity) for online monitoring using appropriate sensors.

### Limitation and future work

Unlike data acquisition with high temporal resolution using commercially available systems, WRAQ and WRAQ-WiFi with 4- and 8-bit counters collect data on a binary counter every 4 and 60 s, respectively, to save battery for long-term recording. The resulting temporal resolution in WRAQ is much lower than in other commercially available systems (e.g., ClockLab from Actometrics Co Ltd.). However, those intervals in WRAQ were set not to exceed the limit of binary counter based on a previous study showing that mice primarily run up to 105 cm/s (Lemieux et al., 2016). However, it is unlikely that 4- and 60-s intervals are too short for long-term analysis over 24 h.

In comparison with the wheel running activity recording system available commercially, in which the data analysis could be done on the same platform using single software (e.g., ClockLab), data analysis in WRAQ system has an additional step to convert the raw data using pipeline python program to match the data format between data acquisition system (Adalogger and Ambient IoT server for WRAQ and WRAQ-WiFi, respectively) and analysis software (ActogramJ). This issue would be resolved by modifying the ActogramJ, which is also open-source software, to enable it to import raw data from WRAQ directly without conversion in the future.

Home cage activity can be measured using the general activity detected by motion sensors (Matikainen-Ankney et al., 2019). Since the running wheel significantly increases the home cage activity, WRAQ/WRAQ-WiFi is likely to affect the home cage activity per se. It would be interesting in the future to measure the general home cage activity detected by motion or capacitive sensors with or without functional WRAQ to address the influence of running wheel access on the home cage general activity.

## Synthesis

Reviewing Editor: Alexxai Kravitz, Washington University in St. Louis Decisions are customarily a result of the Reviewing Editor and the peer reviewers coming together and discussing their recommendations until a consensus is reached. When revisions are invited, a fact-based synthesis statement explaining their decision and outlining what is needed to prepare a revision will be listed below. The following reviewer(s) agreed to reveal their identity: Jeff Jones.

1) The number of mice used for experiments was difficult to determine. Some of the experiments seem to have been run on a single mouse (i.e. chemogenetic activation of the perihabenular area). The number of mice should be clearly stated for all analysis, ideally written right on the relevant figures. Given the ease of the system, the authors should also consider expanding the number of mice in places with only one example mouse, as it will increase confidence in the robustness of the system for readers to see data from more than one mouse.

2) For the experiment in Figure 8 it would be helpful if the authors include an actogram or a summary figure of a control animal that received CNO but did not express the excitatory DREADD. Presumably this control animal would not exhibit a phase delay.

3) A hurdle for many neuroscientists in using open-source behavioral equipment is the analysis of raw data without commercial software. The authors should be commended on presenting python code to analyze wheel running data, yet this code is almost entirely not commented. It would be much more likely to be used if the authors could provide a brief description of each step or function. The authors should also include a more complete description of how to use the code than the included README.md, which is one fragmented sentence. They should also describe how they analyzed the data presented here in the text, and also discuss how this framework compares to other wheel running analyses.

4) The authors were commended for providing the Arduino code. However, it was also not well commented, which will reduce the possibility of users being able to implement or modify it. Please provide commented code, with headings to highlight each major part of the script.

5) The discussion/comparison of WRAQ with current technologies should be expanded to include wheel running as traditional home cage setups. The authors should describe how their technology compares to currently available commercial options.

6) Both reviewers noted that the grammar and language use in many placed was awkward and inhibited reading of the text. The authors should consider a thorough proof-reading to improve the language.

7) Some language should be revised to improve clarify. Specifically, reviewer 1 pointed out:

a. In the abstract, the authors should clarify that WRAQ doesn’t analyze the neural substrate regulating wheel running, it measures wheel running.

b. Line 7 refers to “the conventional cage system", yet there are many rodent caging systems widely in use and not one standard system. The authors should update this language to clarify that WRAQ likely fits in many homecages, with dimensions given so a reader can determine if it fits in their cages.

c. Line 31 refers to home cage activity as “the basis of analysis of behavioral phenotypes", which is not clear what is meant by this.

d. Line 46 refers to “genetically modified mice” as the model to be tested; WRAQ can be used to study wheel running in any mouse line.

e. And in line 47, the language should be changed to describe how researchers using this tool can modify inputs to the mouse condition, whether that’s chemogenetic, optogenetic, or something else in the environment.

8) Some of the figures could be combined into individual panels to create a more concise manuscript.

Minor points:

Figure 4E was truncated and could not be fully viewed.

Figures 1 and 2 and 5 do not seem to be mentioned in the body of the manuscript.

For Figure 3B, can the authors show average wheel running data over the light/dark cycle from all animals?

Please show an enlarged panel of c-fos staining (Fig 8C).

The BOM should be included as a figure in the paper, not as a supplemental excel file.

## Author Response

Responses to the reviewers’ comments

1) The number of mice used for experiments was difficult to determine. Some of the experiments seem to have been run on a single mouse (i.e. chemogenetic activation of the perihabenular area). The number of mice should be clearly stated for all analyses, ideally written right on the relevant figures or included in the figure legends. Given the ease of the WRAQ system to collect data, the authors should consider expanding the number of mice in places with only one example mouse, as it will increase confidence in the robustness of the system for readers to see data from more than one mouse.

To address this point, we performed a novel experiment to examine the effect of chemogenetic activation of the suprachiasmatic nucleus (SCN)-subparaventricular zone (SPZ) instead of perihabenular area (PHb) on the wheel running activity in the revised manuscript. This is because we observed a variable effect of the chemogenetic activation of PHb on wheel running activity in the later analysis, probably because of the possible heterogeneity of PHb differentially targeted by viral vector across trials.

In a novel experiment, we measured the wheel-running activity of a mouse targeted by AAV8-hSyn-hM3D-mCherry to upregulate neuronal activity in the SCN-SPZ upon systemic administration of CNO (new Figure 6A and 6C). Studies showed that SCN-SPZ acted as a master clock and a region relaying the circadian information from the SCN to other brain regions, respectively (Ibuka and Kawamura, 1975; Lu et al., 2001). We measured the wheel-running activity of a mouse targeted by AAV8-hSyn-hM3D-mCherry to upregulate neuronal activity in the SCN-SPZ upon systemic administration of CNO (Figure 6A and 6C) (n = 5 mice). We observed a significant increase of c-Fos-positive cells in the SCN-SPZ following intraperitoneal injection of CNO (Figure 6C). Before CNO injection, the mouse exhibited free-running activity under DD (Figure 6A). Under the guidance of WRAQ-WiFi, injection of CNO at CT14 (red asterisks in Figure 6A) induced a significant shift in the onset of the active period lasting at least 7 days (Figure 6A).

We added this novel result as new Figure 6 and new Results section (p7, l230-l238 of the revised manuscript). We believe that this new result reproducible in the multiple mice will increase confidence in the robustness of the WRAQ system for the readers of our manuscript as the reviewer suggested.

References

Ibuka N, Kawamura H. Loss of circadian rhythm in sleep-wakefulness cycle in the rat by suprachiasmatic nucleus lesions. Brain Res. 1975 Oct 10;96(1):76-81.

Lu J, Zhang YH, Chou TC, Gaus SE, Elmquist JK, Shiromani P, Saper CB. Contrasting effects of ibotenate lesions of the paraventricular nucleus and subparaventricular zone on sleep-wake cycle and temperature regulation. J Neurosci. 2001 Jul 1;21(13):4864-74.

2) For the experiment in Figure 8 it would be helpful if the authors include an actogram or a summary figure of a control animal that received CNO but did not express the excitatory DREADD.

To address this point, we performed a control experiment in which the effect of CNO was administered systemically to the mice expressing GFP in SCN-SPZ. Results showed that intraperitoneal injection of CNO to the mice expressing GFP in the SCN-SPZ did not induce any of the effects on the onset of active period (Figure 6B) and c-Fos in SCN-SPZ (Figure 6D) significantly (n = 5 mice).

This new result was described in new Results section (p7, l238-240 of the revised manuscript).

3) A hurdle for many neuroscientists in using open-source behavioral equipment is the analysis of raw data without commercial software. The authors should be commended for including Python code to analyze wheel running data, yet this code is almost entirely not commented. It will be much more likely to be used by other scientists if the authors could provide a brief description of each step or function. The authors should also include a more complete description of how to use the code than the included README.md, which is one sentence that the reviewers and editor could not understand. A complete markdown file with screenshots of the analysis steps would be ideal. The authors should also describe how they analyzed the data presented here in the text, and also discuss how this framework compares to other wheel running analyses.

To address this point, we provided python program with comments to help the users to understand each step and function in the code. Furthermore, we provided a complete description of the workflow in processing the data from WRAQ in Readme.md file with screenshots of each analysis step. Since we will upload the extended data including the python program to the online repository such as github, extended data in this manuscript will be kept updated reflecting the feedback from the users in the future. Specifically, the raw data was converted into the file readable by ActogramJ, in which the data was supposed to start at zeitgeber time (ZT) 12, by custom-made python program (main_wraq2actj.py provided as new Extended Data, p4, l101-103 of the revised manuscript).

In comparison with the wheel running activity recording system available commercially, in which the data analysis could be done on the same platform using single software (e.g., ClockLab), data analysis in WRAQ system has an additional step to convert the raw data using pipeline python program to match the data format between data acquisition system (Adalogger and Ambient IoT server for WRAQ and WRAQ-WiFi, respectively) and analysis software (ActogramJ). This issue would be resolved by modifying the ActogramJ, which is also open-source software, to enable it to import raw data from WRAQ directly without conversion in the future.

This limitation was also added to the new Discussion section of the revised manuscript (p9, l284-l290).

4) The authors were also commended for providing the Arduino code for their running wheels. However, this code also had very few comments, which will reduce the possibility of users being able to implement or modify it. Please provide commented code, with headings to highlight each major part of the script. Please also include any library dependencies including version numbers.

In the revised manuscript, we intensively revised the Arduino code with additional comments which will help the users to implement or modify it as a part of new Extended Data. In addition, we revised a document which described how to install the dependent libraries with version information and programming environment in a step-by-step manner (“WRAQ_installation.md”). We believe that this will help the users to build their own environment more easily.

5) The discussion/comparison of WRAQ with current technologies should be expanded to include wheel running as traditional home cage setups. The authors should describe how their technology compares to currently available commercial options.

To address this, we rewrote the Discussion section and expanded to compare WRAQ with commercially available options.

Specifically, in comparison with the wheel running activity recording system available commercially, in which the data analysis could be done on the same platform using single software (e.g., ClockLab), data analysis in WRAQ system has an additional step to convert the raw data using pipeline python program to match the data format between data acquisition system (Adalogger and Ambient IoT server for WRAQ and WRAQ-WiFi, respectively) and analysis software (ActogramJ). This issue would be resolved by modifying the ActogramJ, which is also open-source software, to enable it to import raw data from WRAQ directly without conversion in the future.

We also noted in new Discussion section that the size of the WRAQ system (width 5 × depth 5 × height 3.5 inches) is smaller than representative low-profile running wheels (ENV-047, Med Associate Inc., Fairfax, VT, USA; width 6.1 × depth 6 × height 4 inches) used widely in neuroscience studies with mouse models. Since the latter low-profile mouse running wheel was used successfully in standard “shoebox” style individually ventilated cages (e.g., Model #9, Thoren Caging Systems, Inc., Hazleton, PA with width 7.70 × depth 12.17 × height 5.875 inches) (Beeler and Burghardt, 2021; Goh and Ladiges, 2015), it is reasonable to think that our WRAQ fits a wide variety of cage systems.

These paragraphs were added to the new Discussion section (p8, l264-l270 and p9, l284-l290 of the revised manuscript).

6) Both reviewers noted that the grammar and language use throughout was awkward and inhibited reading of the text. The authors should consider a thorough proof-reading to improve the language.

We asked Enago (www.enago.jp) for the English language review to improve the language in the main document as indicated the revised manuscript with highlights. We also proof-read the manuscript to improve the language and correct typos as well.

7) Some language should be revised to improve clarify. Specifically, reviewer 1 pointed out:

a. In the abstract, the authors should clarify that WRAQ doesn’t analyze the neural substrate regulating wheel running, it measures wheel running.

We rewrote this sentence as follows in the revised manuscript (p1, l14-l16).

"Together, these findings indicate that the WRAQ system is a novel and cost effective solution for the analysis of wheel-running activity in mice.”

b. Line 7 refers to “the conventional cage system", yet there are many rodent caging systems widely in use and not one standard system. The authors should update this language to clarify that WRAQ likely fits in many homecages, with dimensions given so a reader can determine if it fits in their cages.

According to the reviewer’s suggestion, we provided detailed information on dimension of the WRAQ and mouse cage we used in the current study in the new Methods section (p2, l61-p3, l65 and p3, l69-l71 of the revised manuscript).

The size of the WRAQ system (width 5 × depth 5 × height 3.5 inches) is smaller than representative low-profile running wheels (ENV-047, Med Associate Inc., Fairfax, VT, USA; width 6.1 × depth 6 × height 4 inches) used widely in neuroscience studies with mouse models. Since the latter low-profile mouse running wheel was used successfully in standard “shoebox” style individually ventilated cages (e.g., Model #9, Thoren Caging Systems, Inc., Hazleton, PA with width 7.70 × depth 12.17 × height 5.875 inches) (Beeler and Burghardt, 2021; Goh and Ladiges, 2015), it is reasonable to think that our WRAQ fits a wide variety of cage systems.

To address the reviewer’s point, we added this to new Discussion to help readers to determine whether WRAQ is applicable to their environment (p8, l264-l270 of the revised manuscript).

References

Goh J, Ladiges W. Voluntary Wheel Running in Mice. Curr Protoc Mouse Biol. 2015 Dec 2;5(4):283-290.

Beeler JA, Burghardt NS. Activity-based Anorexia for Modeling Vulnerability and Resilience in Mice. Bio Protoc. 2021 May 5;11(9):e4009.

c. Line 31 refers to home cage activity as “the basis of analysis of behavioral phenotypes", which is not clear what is meant by this.

We addressed this by rephrasing “the basis of analysis of behavioral phenotypes” with “a basic phenotype” in the revised manuscript (p2, l30).

d. Line 46 refers to “genetically modified mice” as the model to be tested; WRAQ can be used to study wheel running in any mouse line.

To address the reviewers’ comment, we replaced “genetically modified mice” to “mice” in the revised manuscript (p2, l43).

e. And in line 47, the language should be changed to describe how researchers using this tool can modify inputs to the mouse condition, whether that’s chemogenetic, optogenetic, or something else in the environment.

To address the reviewers’ comment, we rewrote this sentence as below to specify how researchers modify inputs to the mouse condition in the revised manuscript (p2, l44-l46).

"Streaming behavioral and environmental signals online would allow researchers to analyze the data in real-time and manipulate the ongoing neural activity at specific circadian times (CT) using genetic and pharmacological interventions.”

8) Some of the figures could be combined into individual panels to create a more concise manuscript.

According to the reviewer’s suggestion, we combined previous Figures 2 and 3 into new Figure 2 in the revised manuscript. We also combined previous Figures 4 and 6 into new Figure 3. This made our manuscript be more concise than before.

Minor points:

1. Figure 4E was truncated and could not be fully viewed. Please fix.

We fixed this problem and presented the entire panel for previous Figure 4E as new Figure 3E in the revised manuscript.

2. Figures 1 and 2 and 5 do not seem to be mentioned in the body of the manuscript. Please address.

We addressed this by referring to the previous Figures 1, 2 and 5 in the new Materials and Methods (p3, l71; p3, l73; p3, l78; p3, l80; p3, l82; p3, l84; p3, l92; p4, l101 of the revised manuscript) and new Results sections (p6, l201 of the revised manuscript).

3. Please show an enlarged panel of c-fos staining (Fig 8C).

In the revised manuscript, we added a magnified view of the panel of c-Fos staining as an inset in new Figure 6C to address this point.
