# Supplementary figures and images for: A Novel Microcontroller-Based System for the Wheel-Running Activity in Mice
Source: eNeuro. 2021 Nov 18;8(6):ENEURO.0260-21.2021. doi: 10.1523/ENEURO.0260-21.2021 (PMC8609968; doi:10.1523/ENEURO.0260-21.2021)

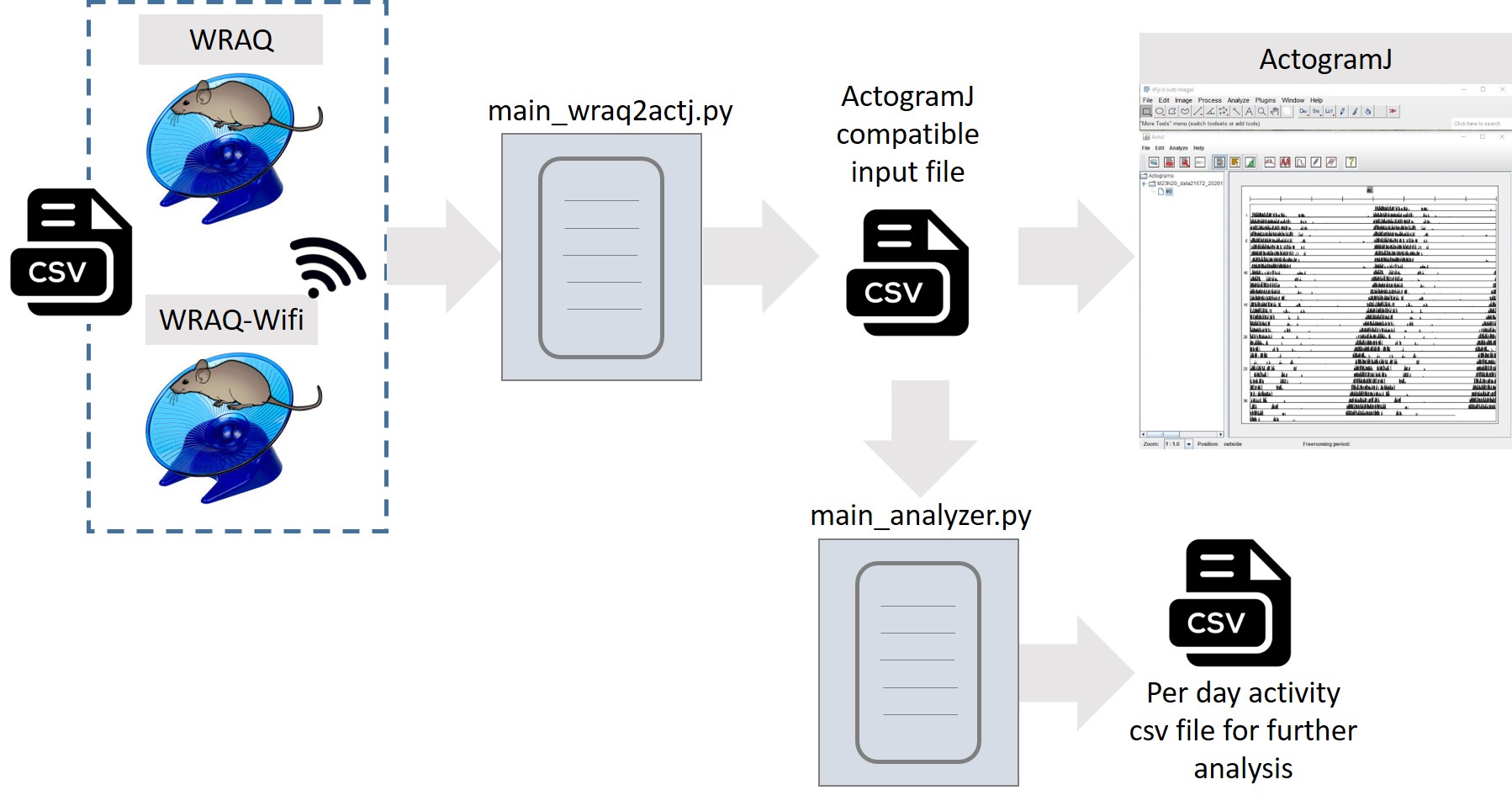

Supplement: Extended Data 1 — Files used in the study. Download Extended Data 1, ZIP file. [file enu-eN-OTM-0260-21-s01.zip › ExtendedData/python/docs/wheelrunner-flow.jpg]

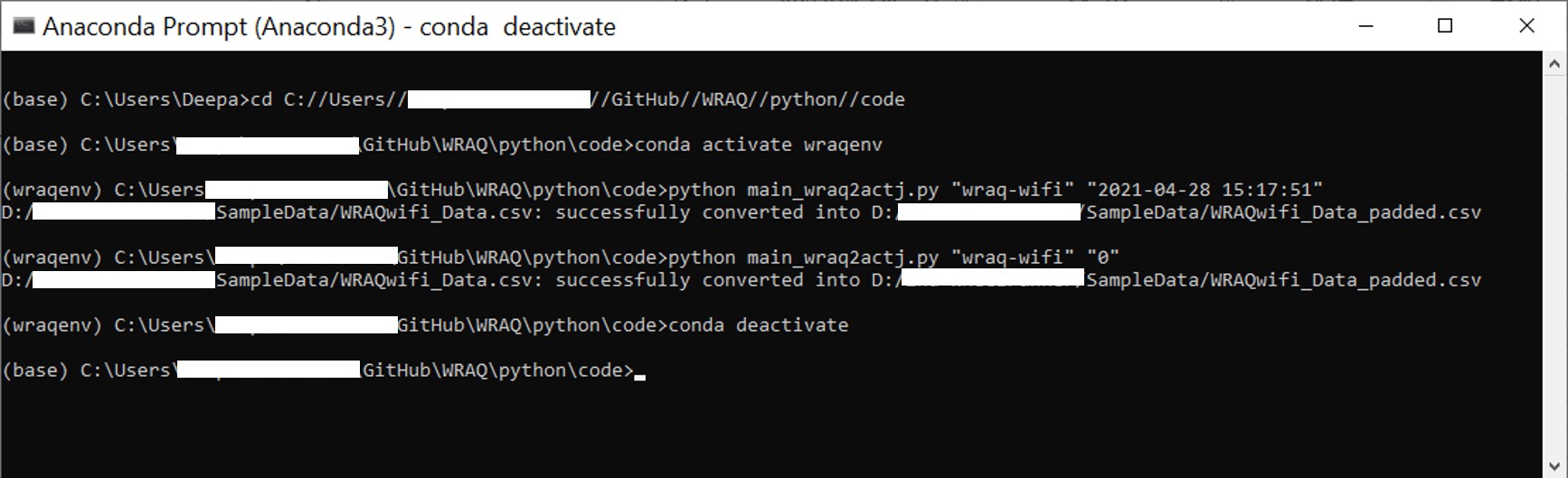

Supplement: Extended Data 1 — Files used in the study. Download Extended Data 1, ZIP file. [file enu-eN-OTM-0260-21-s01.zip › ExtendedData/python/docs/screenshot.jpg]

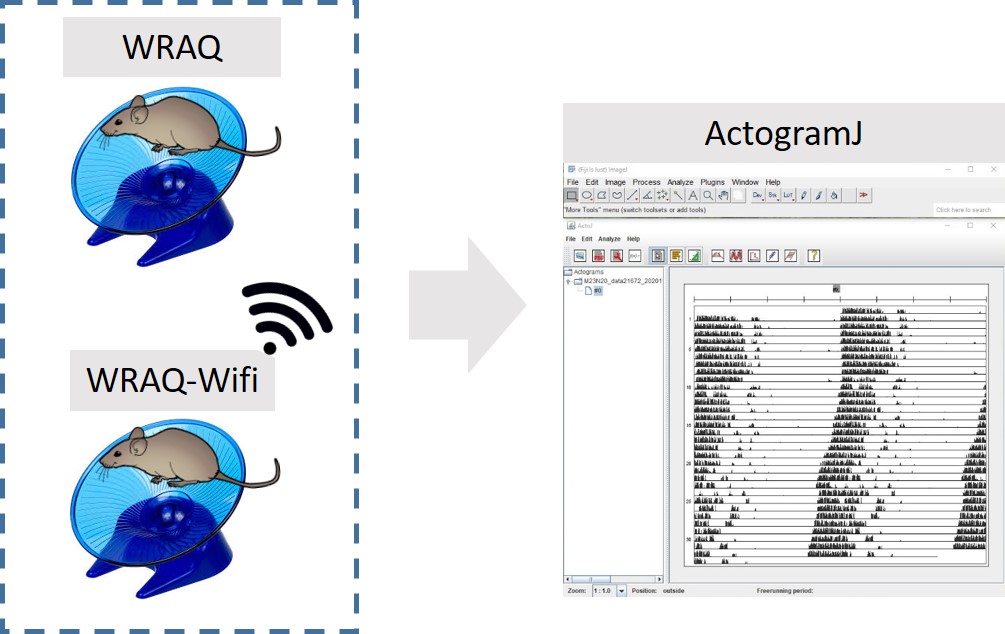

Supplement: Extended Data 1 — Files used in the study. Download Extended Data 1, ZIP file. [file enu-eN-OTM-0260-21-s01.zip › ExtendedData/python/docs/WRAQoverview.jpg]
